# Maternal High Fat Diet-Induced Obesity Modifies Histone Binding and Expression of *Oxtr* in Offspring Hippocampus in a Sex-Specific Manner

**DOI:** 10.3390/ijms20020329

**Published:** 2019-01-15

**Authors:** Kelly A. Glendining, Christine L. Jasoni

**Affiliations:** Centre for Neuroendocrinology, Department of Anatomy, University of Otago, Dunedin 9016, New Zealand; kelly.glendining@otago.ac.nz

**Keywords:** developmental programming, maternal nutrition, neurodevelopment, epigenetic modification, H3K9Ac, H3K9me3, oxytocin receptor, obesity

## Abstract

Maternal obesity during pregnancy increases risk for neurodevelopmental disorders in offspring, although the underlying mechanisms remain unclear. Epigenetic deregulation associates with many neurodevelopmental disorders, and recent evidence indicates that maternal nutritional status can alter chromatin marks in the offspring brain. Thus, maternal obesity may disrupt epigenetic regulation of gene expression during offspring neurodevelopment. Using a C57BL/6 mouse model, we investigated whether maternal high fat diet (mHFD)-induced obesity alters the expression of genes previously implicated in the etiology of neurodevelopmental disorders within the Gestational Day 17.5 (GD 17.5) offspring hippocampus. We found significant two-fold upregulation of oxytocin receptor (*Oxtr*) mRNA in the hippocampus of male, but not female, GD 17.5 offspring from mHFD-induced obese dams (*p* < 0.05). To determine whether altered histone binding at the *Oxtr* gene promoter may underpin these transcriptional changes, we then performed chromatin immunoprecipitation (ChIP). Consistent with the *Oxtr* transcriptional changes, we observed increased binding of active histone mark H3K9Ac at the *Oxtr* transcriptional start site (TSS) in the hippocampus of mHFD male (*p* < 0.05), but not female, offspring. Together, these data indicate an increased vulnerability of male offspring to maternal obesity-induced changes in chromatin remodeling processes that regulate gene expression in the developing hippocampus, and contributes to our understanding of how early life nutrition affects the offspring brain epigenome.

## 1. Introduction

The correct establishment of the epigenome is critical for driving the complex gene expression patterns that underpin normal brain development. Epigenetic regulation via histone modifications, DNA methylation, or non-coding RNA can remodel chromatin structure by changing interactions between DNA and histone proteins, or histone–histone dynamics, to enhance or reduce the accessibility of DNA for the transcriptional machinery. Increasing evidence suggests that these epigenetic mechanisms have a high degree of plasticity during development, and can be modulated by external factors in the in utero environment, including maternal nutrition [1,2]. In this way, the maternal environment may contribute to the programming of offspring disease risk. For example, pregnancies complicated by maternal obesity are associated with a range of poor health outcomes for offspring, including an increased risk of obesity and related metabolic disorders [3]. Animal models of maternal obesity can reliably reproduce the offspring phenotype of elevated obesity risk [4], and recent work from both animal models and human studies indicate that maternal obesity can indeed alter epigenetic regulation of genes related to metabolism and food-seeking behaviors in the brain of offspring [5,6].

Recently, studies have identified obesity during pregnancy also incurs a greater risk for neurodevelopmental disorders in offspring [7,8]. In humans, maternal obesity has been linked to increased incidence of anxiety, depression, learning disabilities, poor psychosocial development, attention deficit hyperactivity disorder (ADHD), and autism spectrum disorders (ASD) [7,8,9,10,11]. Rodent models of maternal obesity during pregnancy have largely reproduced these findings, including elevated risk for impaired social and cognitive behavior in the offspring of obese dams [12,13]. As epigenetic regulation underpins normal developmental gene expression in the brain, and epigenetic abnormalities are linked to the etiology of several neurodevelopmental disorders [14,15,16], the epigenome has surfaced as a potential molecular mediator of the association between maternal obesity and adverse neurodevelopmental programming of offspring. 

When considering how maternal obesity might increase offspring risk for social and cognitive behavioral impairments, changes in the epigenetic regulation of arginine vasopressin (Avp) and oxytocin (Oxt) signaling pathways may be particularly important. These neuropeptides signal via their cognate G protein-coupled *receptors,* arginine vasopressin 1A (Avpr1a) and oxytocin receptor (Oxtr), to modulate diverse aspects of human social behavior, and dysfunction of these systems are well established in neurodevelopmental disorders [17,18,19]. In addition, there is evidence that epigenetic regulation of *Avpr1a* and *Oxtr* expression may be particularly sensitive to environmental disruption during critical periods of development [20,21], and post translational modifications to histone tails is one of the major epigenetic processes regulating the transcription of G-protein coupled receptors in the brain [22]. Late prenatal life (e.g., GD 17.5 in the mouse) is one such critical period where we and others have already observed several changes both to fetal physiology [23] and developmental changes to multiple cell types in multiple brain regions [12,24,25,26,27]. 

Here, we used a mouse model to test the hypothesis that maternal high fat diet (mHFD)-induced obesity programs the risk for neurodevelopmental disorders by altering epigenetic regulation and transcription of *Oxtr* and *Avpr1a* in the offspring brain during late prenatal development. We found that mHFD-induced obesity led to sex-specific programming effects on epigenetic control of gene expression in the offspring brain, with upregulation of *Oxtr* mRNA in the hippocampus of male mHFD offspring associated with increased binding of an active histone mark H3K9Ac at the *Oxtr* promoter. In the hippocampus of female mHFD offspring, *Oxtr* transcription was not altered, but H3K9me3 binding to the Oxtr promoter was decreased. Together, these data reveal sex differences in the vulnerability of offspring to maternal obesity-induced epigenetic programming, resulting in unbalanced expression of a key neuromodulatory signaling receptor in the developing hippocampus.

## 2. Results

### 2.1. Maternal High Fat Diet-Induced Obesity Modulates Expression of Oxtr mRNA in Hippocampus of Male Offspring

**Figure 1:**
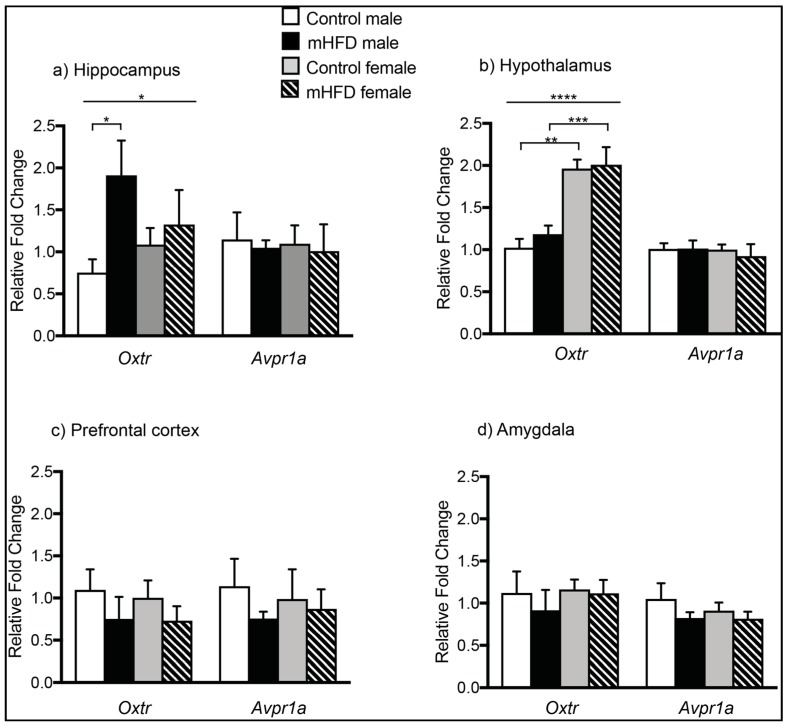
Maternal high fat diet-induced obesity modulates expression of *Oxtr* mRNA in male offspring hippocampus at GD 17.5. Bar graphs depicting mRNA expression of *Avpr1a* and *Oxtr* in maternal high fat diet (mHFD) offspring relative to controls in: (**a**) hippocampus; (**b**) hypothalamus; (**c**) prefrontal cortex; and (**d**) amygdala. Data normalized to reference genes are expressed relative to control males as mean FC ± SEM, with *p* ≤ 0.05 *, *p* ≤ 0.01 **, *p* ≤ 0.001 ***, *p* ≤ 0.0001 ****.

Quantitative PCR analysis indicated that mHFD-induced obesity resulted in male offspring-specific changes in *Oxtr*, but not *Avpr1a* expression, at GD 17.5 (Figure 1a–d). In offspring hippocampus, there was a significant main effect of maternal diet on *Oxtr* mRNA expression (main effect of diet, *F*_(1, 16)_ = 4.85, *p* = 0.0427), with post-hoc comparisons revealing approximately two-fold upregulation of *Oxtr* in male mHFD offspring hippocampus compared to controls (Figure 1a) (*Oxtr*: mHFD males 1.912 ± 0.414, control males 0.753 ± 0.158, *p* = 0.0403). The maternal diet-induced change was specific to mHFD males only, with no differences in hippocampal *Oxtr* expression of mHFD female offspring compared to control females (*Oxtr*: mHFD females 1.087 ± 0.198, control females 1.327 ± 0.411, *p* > 0.05). By contrast, mHFD-induced obesity did not alter hippocampal expression of *Avpr1a* in male or female offspring (*Avpr1a*: mHFD males 1.05 ± 0.08, control males 1.47 ± 0.32; mHFD females 1.01 ± 0.32, control females 1.097 ± 0.22).

Within offspring hypothalamus (Figure 1b), there was a statistically significant main effect of sex on offspring *Oxtr* transcript levels (ANOVA-2; sex *F*_(1, 16)_ = 41.23, *p* < 0.0001), with post-hoc tests revealing significantly higher expression in females versus males at GD 17.5 irrespective of diet (control females 1.96 ± 0.11 vs. control males 1.03 ± 0.102, *p* = 0.0004; mHFD females 2.01 ± 0.21 vs. mHFD males 1.18 ± 0.103, *p* = 0.0012). There was no effect of diet on *Oxtr* expression within the hypothalamus for either sex (ANOVA-2; sex *F*_(1, 16)_ = 0.5616, *p* = 0.4645). No differences were found between groups in hypothalamic levels of *Avpr1a* (mHFD males 1.013 ± 0.098, control males 1.01 ± 0.07; mHFD females 0.923 ± 0.142, control females 1.002 ± 0.06).

In the offspring prefrontal cortex (PFC), no differences in *Oxtr* (*Oxtr*: mHFD males 0.757 ± 0.082, control males 1.14 ± 0.327; mHFD females 0.871 ± 0.232, control females 0.988 ± 0.353) or *Avpr1a* expression were detected among any groups (*Avpr1a*: mHFD males 0.754 ± 0.26, control males 1.097 ± 0.245; mHFD females 0.732 ± 0.172, control females 1.004 ± 0.205) (Figure 1c). Similarly, in the amygdala, there was no effect of maternal diet or offspring sex on expression of *Oxtr* (Figure 1d) (mHFD males 0.92 ± 0.241, control males 1.122 ± 0.254; mHFD females 1.12 ± 0.158, control females 1.16 ± 0.118) or *Avpr1a* (mHFD males 0.824 ± 0.07, control males 1.05 ± 0.185; mHFD females 0.815 ± 0.084, control females 0.912 ± 0.096) (Figure 1d). 

### 2.2. Maternal High Fat Diet-Induced Obesity Alters Histone H3 Acetyl Lysine Binding to the Promoter Region of Oxtr in Hippocampus of Male Offspring

**Figure 2:**
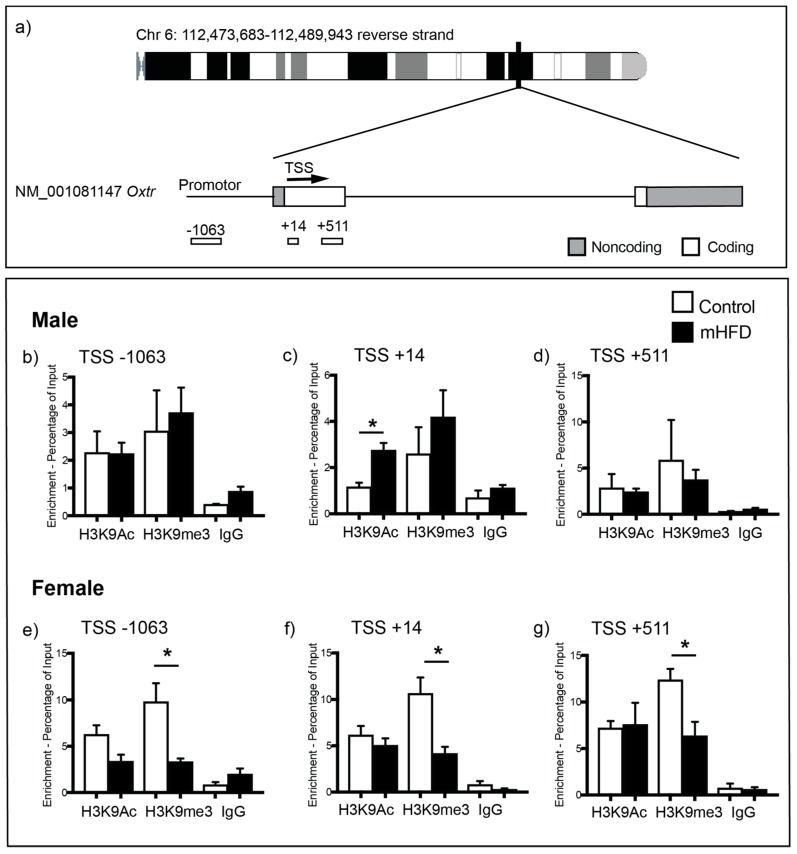
(**a**) ChIP-qPCR primers targeting regions up and downstream of the *Oxtr* transcription start site (TSS). (**b**–**d**) Bar graphs show H3K9Ac, H3K9me3 and IgG (control) binding at the *Oxtr* promoter region of control and mHFD male offspring hippocampus at: (**b**) −1063; (**c**) +14; and (**d**) +501 base pairs from the TSS. (**e**–**g**) Graphs show H3K9Ac, H3K9me3 and IgG (control) binding at the *Oxtr* promoter region of control and mHFD female offspring hippocampus at: (**e**) −1063; (**f**) +14; and (**g**) +501 base pairs from the transcription start site. Data are expressed as mean log2 ChIP/input ± SEM of three independent experiments, as analyzed by unpaired *t*-test, with *p* ≤ 0.05 *.

We next performed ChIP coupled with qPCR to investigate whether increased *Oxtr* gene expression in the hippocampus of male mHFD offspring could be underpinned by altered histone binding within the *Oxtr* promoter region (Figure 2a). We chose to examine acetylation of histone H3 lysine 9 (H3K9Ac) (Figure 2b–d), a marker associated with increased gene expression, and histone H3 lysine 9 trimethylation (H3K9me3) (Figure 2e–g), a marker associated with transcriptional repression, as these have previously been shown to be modulated by maternal nutrition [28,29]. The binding of H3K9Ac at the *Oxtr* promoter was significantly increased in male mHFD offspring hippocampus in comparison to male control offspring, with differential enrichment of H3K9Ac found specifically at the site nearest the transcription start site (TSS) of *Oxtr* (male mHFD 2.75 ± 0.37%; male control 1.16 ± 0.19%; *p* = 0.0123, Figure 2c). There were no differences in levels of H3K9Ac binding to the *Oxtr* promoter region either upstream (Figure 2b) or downstream (Figure 2d) of the *Oxtr* TSS in male hippocampus. In females, maternal diet had no effect on H3K9Ac enrichment at any targeted regions within the *Oxtr* promoter (*p* > 0.05, Figure 2e–g).

We also found sexually dimorphic effects of maternal HFD-induced obesity on H3K9me3 binding at the *Oxtr* promoter. H3K9me3 binding was significantly reduced at all three targeted regions in mHFD female offspring hippocampus in comparison to control females (Figure 2e–g) (−1063; female mHFD 3.32 ± 0.34%; female control 9.795 ± 2.00%; *p* = 0.0332; +14; female mHFD 4.16 ± 0.72%; female control 10.65 ± 1.72%; *p* = 0.0251; +511; female mHFD 6.36 ± 1.51%; female control 12.38 ± 1.19%; *p* = 0.0351). No differences between male mHFD and control offspring were observed for H3K9me3 binding (Figure 2b–d). 

Together, these observations are consistent with the idea that sex-specific effects of maternal diet on offspring *Oxtr* gene expression in the hippocampus may be underpinned by maternal diet-induced epigenetic change.

## 3. Discussion

We provide evidence that in utero exposure to mHFD-induced obesity alters *Oxtr* gene expression and histone binding at the *Oxtr* promoter in offspring hippocampus in a sexually dimorphic manner. In response to mHFD, *Oxtr* transcription increased and H3K9Ac binding was enriched at the *Oxtr* promoter in male offspring hippocampus. These observations are consistent with current understanding of the function of histone acetylation at the gene promoter, which typically correlates with transcriptional activation [30], including that of G-protein coupled receptors in the brain [31]. By contrast, mHFD was associated with decreased binding of the repressive histone mark H3K9me3 to the *Oxtr* promoter in female offspring hippocampus, with no downstream changes in *Oxtr* transcript levels. 

The mHFD-induced changes in *Oxtr* mRNA levels concomitant with altered histone binding at the *Oxtr* promoter in male offspring hippocampus indicate that *Oxtr* transcriptional control is deregulated as a consequence of maternal diet-induced obesity. This altered *Oxtr* gene expression is likely to have downstream functional consequences on oxytocinergic signaling within the hippocampus, impacting hippocampal development and later-life function. Oxt surges in the maternal circulation in late gestation, in preparation for parturition and subsequent lactation [32]. In addition to modulating maternal behavior, elevated Oxt has been shown to reach the fetal brain where it plays an integral role in a number of processes related to the formation of neuronal circuitry and synaptic transmission within the hippocampus. Oxt signaling is important for the late gestation/early neonatal switch in GABAergic signaling from excitatory to inhibitory [33], and perturbations have been shown to lead to seizures [34]. Oxt signaling is also important for the maturation of the glutamatergic system within the hippocampus, through its ability to modulate dendrite branching and synapse development, synaptic transmission, and the synchronicity of neuronal networks [35]. Together, these data paint a picture wherein late gestational Oxt/Oxtr signaling is vital for the development of the balance of GABAergic and glutamatergic neural circuitry and transmission within the offspring hippocampus; and situations in which this is altered, such as maternal obesity, it would be expected to lead to hippocampal dysfunction. Indeed, maternal obesity is associated with altered GABAergic signaling in the developing hippocampus of offspring [36], impaired growth, cell division and neurogenesis [37], and hippocampal specific changes in expression of genes involved in synaptogenesis [38]. Thus, given the dependence of hippocampal neural circuitry development on Oxtr signaling, it seems likely that the epigenetic dysregulation of *Oxtr* expression reported here may underpin at least some of these deficits observed in the hippocampus of mHFD offspring. 

Oxytocin signaling via the Oxtr is strongly implicated in modulating social behavior. Disrupted Oxtr function is implicated in a number of neurodevelopmental disorders, particularly those associated with core social deficits [39,40], and abnormal social behavior can be induced by modulating oxytocin systems in animal models [41]. Recent animal studies also suggest that some of the cognitive difficulties associated with ASD may result from disrupted hippocampal function and circuitry [42,43], and disrupting hippocampal-cortical signaling results in ASD-like behaviors [44]. The mHFD-induced deregulation of Oxtr signaling in the hippocampus of male offspring may therefore also have functional outcomes in terms of social impairments. Consistent with this, animal models of maternal obesity have reported social deficits in offspring [38,45], as well as altered Oxt circuitry [46]. 

Although limited in number, studies examining sex differences in offspring brain in response to maternal obesity indicate divergent developmental vulnerabilities might exist between the sexes. For example, male offspring are more susceptible to mHFD-induced changes in brain gene expression [47], and to deregulation of epigenetic regulators in the hippocampus, amygdala and PFC [12]. These sex-differences in vulnerability to mHFD-induced changes in the brain may contribute to the known male bias in the prevalence of some neurodevelopmental disorders such as ASD and ADHD [48,49]. 

It is unclear why changes to H3K9me3 binding at the *Oxtr* promoter of mHFD females were not predictive of gene expression. There is growing consensus that histone lysine methylation is interrelated with DNA methylation, reviewed in [50]. Decreased H3K9me3 binding at the *Oxtr* promoter in mHFD females might therefore reflect changes in methylation, however previous studies have found changes in methylation of the *Oxtr* promoter directly correlate with transcription, at least in the adult brain [17,51]. Thus, histone lysine methylation may be more important for transcriptional control of *Oxtr* in an adult rather than developmental context. Extensive examination of the transcriptional regulation of the *Oxtr* gene across development and into adulthood would provide clarity here. 

Finally, the molecular changes that account for the link between maternal obesity and epigenetic changes remain obscure. There is a complex relationship between inflammatory cytokine signaling and histone deacetylase activity [52,53], thus the expression or activity of enzymes that catalyze epigenetic changes may be modulated by mHFD-associated gestational inflammation [23]. More work in the area of how histone modifying enzymes are regulated to affect epigenetic change would shed important light on this. 

With growing evidence that maternal obesity is associated with an increased risk of offspring neurodevelopmental disorders [7,54,55,56,57], and an increasing global prevalence of pregnancies complicated by maternal obesity [58,59], understanding the impact of maternal obesity on epigenetic regulation of brain development will be critical for developing future interventions to protect the offspring brain. 

## 4. Materials and Methods

### 4.1. Animal Housing and Maternal Diet

All experiments were performed using protocols approved by the Animal Ethics Committee of the University of Otago (ET 16/17; 19 February 2018), and in accordance with the guidelines of the National Animal Welfare Advisory Committee, NZ. Generation of the mHFD-induced obesity mouse model has been previously described in [12,27]. Briefly, 4-week-old female C57/BL6 mice were housed under a 12 h light–dark cycle at constant temperature (21 ± 2 °C), and randomly allocated to an ad libitum control diet (10% kcal fat; D12450B, Research Diets, NJ, USA), or a high-fat diet (45% kcal fat; D12451, Research Diets, NJ, USA). Respective diets were maintained for 8 weeks, and through mating and gestation. Bodyweight was monitored weekly, and only high-fat diet fed animals determined significantly heavier than controls (≥30%, *t*-test, *p* ≤ 0.05) were used to generate offspring. Females were timed-mated with 4-month-old males maintained on chow. Litters were collected by caesarean at GD 17.5 between the 09:00 and 10:00. Sex was determined by *Sry* PCR [12]. 

### 4.2. RNA Extraction

Brains were cryosectioned at 70 μm, and incubated in RNAlater-ICE (Thermo Fisher Scientific, Melbourne, Australia) at −20 °C overnight. Hippocampi were micro-dissected from sections using an Olympus SZ61 microscope and RNA extracted with ZR-Duet DNA/RNA MiniPrep kit (Zymo Research, Irvine, USA), and treated with DNase I. RNA purity was assessed by NanoDrop ND-1000, quantified using a Qubit 2.0 Fluorometer, and reverse transcribed with Quanta qScript XLT cDNA SuperMix. 

### 4.3. Quantitative PCR (qPCR)

All qPCR assays were performed on the Viia7 Real-Time PCR System. Triplicate (10 µL) reactions contained 2× PowerUp SYBR Green, 1 µL cDNA and 250 nm forward and reverse primers. Each qPCR run included no template-control and RNA as template reactions, and dissociation analysis to ensure amplification specificity. Primers were designed as previously described [27,60], with sequences and Genbank Accession numbers listed in Table S1. Previously validated region-specific reference genes with stable expression irrespective of maternal diet were used (*Gapdh*, *Pgk1*, *Tbp*, *Sdha*) [12]. For each group, fetal tissue was gathered from *n* = 5 independent adult female pregnancies. For each pregnancy, fetal tissue from 4 fetuses per sex was pooled. Thus, each group consisted of data derived from tissue from 20 fetuses (4 per pregnancy × 5 pregnancies). Normalized fold change in comparison to control males was determined using 2^−ΔΔ*C*T^ with efficiency correction [61]. 

### 4.4. ChIP-qPCR

Dissected hippocampal tissue was crosslinked in 1% formaldehyde for 10 min, quenched with glycine [0.125 M], and stored at −80 °C until required. ChIP validated rabbit polyclonal antibodies [anti-H3K9me3 (Abcam, ab8898), anti-H3K9Ac (Abcam, ab4441), and preimmune IgG (Abcam, ab1220)] were conjugated to protein G Dynabeads in 0.5% BSA for 3 h at 4 °C. Chromatin was enzymatically sheared using Micrococcal Nuclease, extracted, and lysate was precleared for 1 h at 4 °C. Chromatin was immunoprecipitated with 2 µg of antibody overnight at 4 °C. Immunoprecipitated DNA was washed from Dynabeads, eluted in TE, reverse crosslinked for 4 h at 65 °C, and column purified. All ChIP-qPCR primer sequences are listed in Table S2. Animal numbers and study design were as in 4.3 qPCR above.

### 4.5. Statistical Analysis

Statistical analyses were performed using Prism (v7.0d) software. For gene expression qPCR, average fold change relative to control males was determined using the 2^−ΔΔ*C*T^ relative quantification method with efficiency correction [61], using a two-way ANOVA (diet × sex), followed by Bonferroni post hoc pairwise comparisons. For ChIP-qPCR, data were normalized to input chromatin using the ∆*C*_T_ method, and analyzed by Student’s unpaired *t*-test. Results were considered statistically significant where *p* ≤ 0.05. 

## Supplementary Materials

Supplementary Materials can be found at http://www.mdpi.com/1422-0067/20/2/329/s1.

## Abbreviations

ADHD	attention deficit hyperactivity disorder
ASD	autism spectrum disorders
Avp	Arginine vasopressin
Avpr1a	Arginine vasopressin receptor 1A
ChIP	Chromatin Immunoprecipitation
GD	Gestational day
H3K9Ac	Histone 3 Lysine 9 Acetylation
H3K9me3	Histone 3 Lysine 9 methylation
mHFD	Maternal high fat diet
Oxt	Oxytocin
Oxtr	Oxytocin receptor
qPCR	Quantitative PCR
TSS	Transcription start site
