# Supplementary material for: Maternal High Fat Diet-Induced Obesity Modifies Histone Binding and Expression of Oxtr in Offspring Hippocampus in a Sex-Specific Manner
Source: Int J Mol Sci. 2019 Jan 15;20(2):329. doi: 10.3390/ijms20020329 (PMC6359595; doi:10.3390/ijms20020329)
Supplement: Supplementary file 1 [file ijms-20-00329-s001.pdf]

Supplementary Materials

Table S1: Primer sequences used for qPCR analysis of gene expression.

**Gene Symbol**	**Accession no.**	**Forward Primer 5"-3"**	**Reverse Primer 5"-3"**
Avpr1a	NM_016847.2	TTCGTTTGGACCGATTCCGA	CGTCCCAGTGCTGTTTGTTG
Oxtr	NM_001081147.1	TGGTTTGCATCCCTCCTGTC	AGGTGTGCCGTCTTTCACAA
Gapdh	NM_001289726.1	AACTTTGGCATTGTGGAAGG	ACACATTGGGGGTAGGAACA
Pgk1	NM_008828.3	CTCCGCTTTCATGTAGAGGAAG	GACATCTCCTAGTTTGGACAGTG
Sdha	NM_023281.1	GATTACTCCAAGCCCATCCA	GCACAGTCAGCCTCATTCAA
Tbp	NM_013684.3	GAAGAACAATCCAGACTAGCAGCA	CCTTATAGGGAACTTCACATCACAG

Table S2: Primer sequences used for ChIP-qPCR analysis of histone binding to the *Oxtr* promoter.

Genomic Sequence; NC_000072.6 Reference GRCm38.p4 C57BL/6J

**Target Region**	**Forward Primer 5"-3"**	**Reverse Primer 5"-3"**
Oxtr TSS -1063	TGGATTCTCCGCCCAGATTG	GAGTTTGCAAAGACGCCGAG
Oxtr TSS +14	AGTATGAGACACAGGACCGC	CGCAGCCAACTGGAGTATCG
Oxtr TSS +511	CAGGGCTGGATGAAGACCG	AGGTGCACATTTTCTCGCTG
